# Familial Risk Factors in Relation to Recurrent Depression Among Former Adolescent Psychiatric Inpatients

**DOI:** 10.1007/s10578-021-01146-1

**Published:** 2021-03-02

**Authors:** Joonas Halonen, Helinä Hakko, Kaisa Riala, Pirkko Riipinen

**Affiliations:** 1 Research Unit of Clinical Neuroscience, Psychiatry, University of Oulu, P.O.BOX 5000, 90014 Oulu, Finland; 2 Department of Psychiatry, Oulu University Hospital, Oulu, P.O.BOX 26, 90029 Oulu, Finland

**Keywords:** Adolescent, Family-related factors, Psychiatric disorders, Recurrent depression, Register-based follow-up study

## Abstract

Treating recurrent depression is a challenge for clinical practitioners. We investigated which family environmental factors contribute to differences between recurrent and non-recurrent depression by the young adulthood of the former adolescent inpatients. The initial sample covered 237 adolescent psychiatric inpatients with depression, of which 35.4% had later diagnosed with recurrent depression. Recurrence in depression was associated to distant maternal relationships in both male (p = 0.022) and female patients (p = 0.042). In females, the likelihood for recurrent depression was also related to psychiatric problems of the father (p = 0.013) and siblings (OR = 3.7, p = 0.032), and having a grand multiparous mother (p = 0.005). Our results emphasise the need for effective family-centred approaches in treatment of adolescents with depression.

## Introduction

Depression is a significant and growing global health problem. It is estimated that approximately one fifth of Finnish people will have a depressive episode at least once during their lifetime and, in over half of them, their depression will be recurrent [1]. The prevalence of depression in childhood does not differ between genders, but, in adolescence, the gender gap gradually grows, so that adult women are nearly twice as likely compared to adult men to be diagnosed with depression [2, 3]. Depression is associated with significant negative consequences, such as psychosocial disadvantages, decreased enjoyment of life, somatic health problems and increased mortality. At the beginning of the 21th century, the World Health Organization (WHO) estimated that unipolar depression was the most common, and bipolar depression the 6^th^ most common cause of disability in people aged between 15 and 44 years [4].

Depression can occur at any age, but the first depressive episode is often experienced during adolescence [5]. The precise mechanism of depression is not known, but the common consensus is that it is a combination of genetic factors, behavioural and cognitive models learned from parents, and environmental stress factors, such as loss of a relative or onset of a serious disease, which may mould neurochemical pathways in the brain and trigger a depressive episode [6].

### Effect of Family Background in Depression

The family environment plays an essential role in the development of depression among children and adolescents, since children spend most of their time with their parents and other family members. Childhood family instability is commonly linked to offspring’s later mental health problems [7]. Parental mental health problems, especially those of mothers, are also associated with an increased risk of adolescent depression [8, 9]. Other predisposing factors for offspring’s depression are shown to include: single-parenthood, parental low education or unemployment and childhood sexual abuse [10–15]. Researchers have described four possible mechanisms for a parent’s depression contributing to the onset of depression in their offspring: (1) genetic heritability, (2) innate dysfunctional neuro-regulatory mechanisms on the child, (3) child exposure to negative parenting patterns, and (4) stressful circumstances in a child’s life [16].

Exposure to Adverse Childhood Experiences (ACEs), such as having a depressed family member, has shown to have a negative effect on adolescent mental health [17]. Moreover, a depressed family member, especially the mother, can lead to other adverse experiences such as child neglect and abuse or parental substance abuse [8]. According to Shonkoff and workgroup [18], these experiences can generate toxic stress for a child, which, when accumulated, can increase the risk of childhood depression. The researchers of that study suggested that ACEs can disrupt the neurochemical circuitry in the brain, and other metabolic and/or organ functions, during sensitive developmental periods, leading to impairment of memory and learning functions. Further, ACEs can also increase the levels of inflammatory markers in children, which, in turn, can have long-lasting effects to health. This includes increasing the risk of depression, but also of somatic illnesses, such as cardiovascular diseases and asthma.

Long-lasting harmful parenting patterns can negatively affect a child’s developing brain architecture and cause long-lasting effects on a child’s behaviour and overall stress response system. Therefore, treatment of a mother’s depression does not necessarily translate to improving the mental health of their child [19]. One example of this is when depressed mothers have more difficulties in recognizing and reading their child´s happy faces and gestures, which might, in turn, lead to a decrease in responses to these gestures by the mothers. Thus, a child’s ability to learn of how to communicate their feelings may be hindered and skewed [20].

### Recurrent Depression

As summarised in the research paper by Pettit and colleagues [21], recurrent depression is defined by a person having subsequent depressive episodes after the initial episode and a symptomless remission period of approximately eight weeks. However, this definition leaves open what “symptomless” period and its length mean. They noted that the proposed mechanisms of recurrence in depression generally fall in to two categories, namely the “scar model” and the “liability model”. In the scar model, the process of having a depressive episode somehow changes the architecture of the brain in a way that increases the probability of a new episode. In the liability model, the patient has, even before their first episode of depression, one or more underlying risk factor which directly increase their chances of having a depressive episode, providing the underlying factor is present [21].

In previous studies, many patients are reported to fail to achieve complete recovery between successive episodes of depression [22]. The studies have also suggested that sub-acute inter-episodic symptoms associate with shorter asymptomatic periods, more rapid and increased rates of relapse and recurrence, and a greater number of, and more chronic, major depressive disorder (MDD) episodes [23], as well as with significant social and occupational impairment [24]. This partial remission is often characterized by the presence of symptoms such as depressed mood, anxiety, somatic symptoms and sexual dysfunction, without meeting the full criteria for MDD [24]. In recurrent depression, the second episode usually occurs within five years of the first episode, regardless of the age group (children, adolescents and adults) of patients [22]. Individuals with recurrent MDD have shown to experience an average of five separate depressive episodes during their lifetime [25].

Pettit and colleagues [21] analysed multiple possible factors relating to recurrence in depression and showed that adolescent’s own history of depression, as well as parental recurrent depression, were the most notable predictors for recurrent depression in adolescents. The review by Burcusa [26] and Iacono reported that additional risk factors for recurrent depression are the age at onset of first depressive episode, the number of previous episodes, the severity of the first episode and other psychopathological comorbidity. Of these risk factors, the latter two were only associated with adult recurrent depression [26].

Using the data of the Minnesota Twin Family Study, Wilson and workgroup [22] examined the effects of several premorbid risk factors of MDD of offspring aged 11 years. They reported that parental depression and antisocial behaviour, in association to the offspring’s negative emotionality and disconstraints, externalizing symptoms, and childhood related physical abuse, assault and maltreatment increased the offspring’s risk for early-onset depression (compared to late-onset depression) in both recurrent and non-recurrent subtypes of depression. In their study, notable risk factors for recurrent depression in offspring, compared to offspring with single-episode depression, included: lower positive emotionality, higher trait anxiety and childhood sexual abuse/assault, but not parental psychiatric disorders. The researchers summarised that risk of offspring recurrent depression was largely a function of its earlier onset of the disorder. In an adolescent study, previous history of MDD, dysphoric mood and dysfunctional thinking patterns were stronger predictors of recurrent episodes than to first onset episodes and, conversely, major stressful life events were more likely associated with first onset episodes than recurrent episodes [27].

### The Present Study

The purpose of this study was to investigate the association of several familial risk factors with recurrence of depression, among former adolescent psychiatric inpatients diagnosed with depression at ages 13 to 17 years. By recognizing early enough the adolescents at high-risk of developing recurrent depression, we could allocate clinical resources more efficiently to this patient group and, thus, decrease risk for recurrence in depression of this vulnerable group of patients. Based on findings in the earlier literature, we hypothesised that poorer family conditions and exposure to stressful family environments in childhood make it more likely that children and adolescents will experience a recurrent path of depression.

## Methods

### Study Sample

This study is a part of an ongoing clinical follow-up project, which investigates psychosocial risk factors and long-term outcomes of psychiatric inpatients hospitalized between the ages of 13–17 years old. The original study population includes 508 adolescents (208 boys, 300 girls) admitted to acute psychiatric inpatient care in Oulu University Hospital in Finland, between April 2001 and May 2006 (later referred as index hospitalization). The catchment area of the hospital covers the whole of Northern Finland, accounting for 43% of Finland’s total geographical area. The study population was homogenous, with Caucasian ethnicity accounting for 98% of the population.

Both the adolescents and at least one parent’s (or guardian’s) signed informed consent was required before participation in this study was allowed. Subjects who were aged over 18 years at admission, mentally retarded, had organic brain disorders or did not provide written informed consent for participation were excluded from the study. Participation rate in the study was high, with 83.7% of the eligible adolescents (n = 637) included. The study protocol was approved by the Ethics Committee of University of Oulu, Finland.

### Definitions of Recurrent and Non-Recurrent Depression

The information on treatment episodes for depression of the study subjects was obtained from the National Finnish Care Register for Health Care (CRHC) until to the end of 2016. Information on inpatient treatments covered the whole life time of study participants, while data on outpatient visits to specialised level of health care was available from 1998 onwards. Depending on the year of data entry, the follow-up time after the index hospitalisation (during years 2001–2006) to the end of year 2016 varied between 10 and 15 years.

The definition of depression was based on the International Statistical Classification of Diseases and Related Health Problems, 10^th^ revision. According to the ICD-10, a depressive episode is classified as a minimum of two week period of almost constantly having two or more of the following symptoms: decreased mood, increased fatigue or decreased interest in previously enjoyable activities. Moreover, a patient has to have one or more of the following symptoms so that the total number of symptoms is four or more: excessive guilt, lowered self-confidence, recurrent thoughts of death, difficulties concentrating, agitation or retardation, changes in eating or in weight, difficulties sleeping, suicidal behaviour or attempted suicide. Depression is defined as being recurrent if the patient has two or more separate depressive episodes separated by at least two symptom free months [26].

For the purpose of our study, 272 study subjects with hospital-diagnosed depression (ICD-10: F3-codes) were identified from CRHC by the end of 2016. Those participants who had been diagnosed with bipolar disorder (ICD-10: F30, F31, F34.0) (n = 30) or schizophrenia spectrum disorder (ICD-10: F20, F21, F25) (n = 5) were excluded from the current study sample.

The final sample of the current study sample consisted of 237 patients with a diagnosis of depression over their lifetime. Of these, 84 (35.4%) patients had developed recurrent depression and 153 (64.6%) had only one depressive episode. The mean (SD) age at the onset of first depressive episode was 16.4 (3.1) years in recurrent and 15.8 (2.4) years in the non-recurrent depression group.

### Adolescence-Related Characteristics

#### Psychiatric Disorders During Adolescence

Patients were interviewed during their index hospitalization between the ages of 13–17, using the semi-structured Finnish version of the Schedule for Affective Disorder and Schizophrenia for School-Age Children Present and Lifetime (K-SADS-PL) [28]. The K-SADS-PL defines psychiatric disorders according to the Diagnostic and Statistical Manual of Mental Disorders, IV revision (DSM-IV), which was further converted to ICD-10 diagnostic classification. The psychiatric disorders were categorised as follows: (1) Affective disorders (F3, excluding F30.2 and F32.3) (2) Substance use disorders (F10-F19) (3) Anxiety disorders (F4, excluding F43.2) (4) Behavioural disorders (F90-92, excluding 91.3) and (5) Psychotic disorders (F2, including F30.2 and F32.3). In the current study, a total of 59% (n = 140) of the study participants (n = 237) had adolescent psychiatric disorders belonging to at least two different psychiatric diagnostic groups, the rate being 57% (n = 41) in males and 60% (n = 99) in females. This co-morbidity rate did not differ between recurrent and non-recurrent depression groups either in males (50%, n = 11 vs. 60%, n = 30) (χ^2^ = 0.62, df = 1, p = 0.430) or in females (66%, n = 41 vs. 56%, n = 58) (χ^2^ = 1.56, df = 1, p = 0.212).

#### Family-Related Factors

Information on family type, family size and birth order was based on the K-SADS-PL. Family type (i.e. home environment preceding admission to adolescent psychiatric inpatient care) was split into the following categories: two biological-parent family; one biological-parent family, single-parent family and blended family (i.e. mother or father with cohabiting partner); child welfare placement (i.e. children’s home or family community home); and other home environment (i.e. foster family, living alone, or residential home). The information about the family size (indicating a grand multiparous mother) was dichotomised as having less than or over five children in the primary family. The birth order of the study participant was categorized as: only child, oldest child (first born), middle-born and youngest child (last born).

The following family-related factors were obtained from the European Addiction Severity Index (EuropASI) interview [29], performed during the index hospitalization period. Educational level of a parent involved the categories: low (no studies completed after compulsory school, not known), middle (student, vocational courses, or vocational qualification) and high (polytechnic, university) level of education. Employment status of a parent indicates whether a mother or father has been in part- or full-time work during the last 3 years (yes, no). Information on parental psychopathology was gathered by asking whether the adolescent perceived that somebody in their primary family (mother, father, siblings) would have needed care (yes, no) due to their psychiatric or substance use related problems. Distant relationship with family members was based on the question inquiring “Would you say (yes, no) you have had close long-lasting relationship with your mother/father/siblings?”. Information on parental death was available at dichotomized level (yes, no).

### Statistical Analyses

Statistical significance of group differences in categorical variables was assessed with the chi-square or Fisher’s Exact test, and in continuous variables, with Student’s *t* test or Mann–Whiney *U* test. A logistic regression analysis was used to examine the association of family-related factors (Model 1: family type, level of maternal/paternal education, employment status of mother/father, death of a parent, large family size, sibling status), perceived characteristics of the family members (Model 2: distant relationship with mother/father/siblings, psychiatric problems of mother/father/siblings, substance use problems of mother/father/sibling) and adolescent psychiatric disorders (Model 3: affective disorders, substance use disorders, conduct disorders, anxiety disorders, psychotic disorders) to likelihood of recurrence of depression by young adulthood. Model 4 includes all variables presented in Tables 1, 2, 3 as potential predictors for recurrence of depression. In all models, the variables were entered to the stepwise logistic regression model using forward selection criteria and Table 4 reports the results of final models showing statistically significant association with outcome variable (recurrence of depression). All statistical tests were two-tailed and the limit for statistical significance was set at p ≤ 0.05. The statistical software used in analyses was IBM SPSS, version 25.

**Table 1:** Family-related factors in childhood and adolescence of study subjects, according to recurrence of depression by young adulthood.

	Total data (n = 237)	Males			Females		
Family-related factors in adolescence of study subjects		Recurrent depression (n=22)	Non-recurrent depression (n= 50)	Group difference, *p*	Recurrent depression (n=62)	Non-recurrent depression (n= 103)	Group difference, *p*
Family type, *n (%)*				0.570			0.297
Two biological parents	85 (35.9)	5 (22.7)	10 (20.0)		21 (33.9)	49 (47.6)	
One biological parent	77 (32.5)	6 (27.3)	22 (44.0)		20 (32.3)	29 (28.2)	
Child welfare placement	36 (15.2)	6 (27.3)	10 (20.0)		8 (12.9)	12 (11.7)	
Other home environment	39 (16.5)	5 (22.7)	8 (16.0)		13 (21.0)	13 (12.6)	
Level of maternal education, *n (%)*				0.724		ss	0.900
Low	115 (48.5)	13(59.1)	25 (50.0)		29 (46.8)	48 (46.6)	
Middle	73 (30.8)	5 (22.7)	12 (24.0)		22 (35.5)	34 (33.0)	
High	49 (20.7)	4 (18.2)	13 (26.0)		11 (17.7)	21 (20.4)	
Level of paternal education, *n (%)*				0.642			0.708
Low	126 (53.2)	11 (50.0)	26 (52.0)		36 (58.1)	53 (51.4)	
Middle	80 (33.8)	9 (40.9)	16 (32.0)		19 (30.6)	36 (35.0)	
High	31 (13.1)	2 (9.1)	8 (16.0)		7 (11.3)	14 (13.6)	
Employment status of a parent, *n (%)*							
Mother not at work	92 (38.8)	12 (54.5)	19 (38.0)	0.192	25 (40.3)	36 (35.0)	0.489
Father not at work	87 (36.7)	11 (50.0)	21 (42.0)	0.529	26 (41.9)	29 (28.2)	0.069
Death of a parent*, n (%)*	19 (18.0)	4 (18.2)	7 (14.0)	0.726	5 (8.1)	3 (2.9)	0.153
Large family size (five or more siblings)*, n (%)*	13 (13.1)	5 (22.7)	5 (10.0)	0.265	14 (22.6)	7 (6.8%)	0.003**
Sibling status (birth order), *n (%)*				0.909			0.056
Only child	37 (15.6)	5 (22.7)	14 (28.0%)		5 (8.1)	13 (12.6)	
Oldest child	55 (23.2)	4 (18.2)	10 (20.0%)		11 (17.7)	30 (29.1)	
Middle-born	64 (27.0)	7 (31.8)	12 (24.0%)		24 (38.7)	21 (20.4)	
Youngest child	81 (34.2)	6 (27.3)	14 (28.0%)		22 (35.5)	39 (37.9)	

^*^p < 0.05

^**^ *p* < 0.01

^***^ *p* < 000.1

**Table 2:** Perceived characteristics of family members (mother, father, siblings) of the study subjects, by recurrence of depression by young adulthood.

		Male			Female		
Perceived characteristics of family members	Total data (n = 237)	Recurrent depression (n = 22)	Non-recurrent depression (n = 50)	Group difference, *p*	Recurrent depression (n = 62)	Non-recurrent depression (n = 103)	Group difference,*p*
Maternal characteristics*, n (%)*							
Distant relationship with mother, yes	64 (27.0)	10 (45.5)	11(22.0)	0.044*	21 (33.9)	22 (21.3)	0.076
Psychiatric problems, yes	26 (18.2)	1 (4.5)	6 (12.0)	0.427	9 (14.5)	10 (9.7)	0.349
Substance use related problems, yes	33 (13.9)	4 (18.2)	3 (6.0)	0.190	9 (14.5)	17 (16.5)	0.734
Paternal characteristics*, n (%)*							
Distant relationship with father, yes	110 (46.2)	12 (54.5)	18 (36.0)	0.141	32 (51.6)	48 (46.6)	0.533
Psychiatric problems, yes	24 (10.1)	0 (0.0)	2 (4.0)	1.000	13 (21.0)	9 (8.7)	0.025*
Substance use problems, yes	63 (26.6)	2 (9.1)	14 (28.0)	0.123	22 (35.5)	25 (24.3)	0.122
Sibling characteristics*, n (%)*							
Distant relationship with sibling(s), yes	52 (21.9)	8 (36.4)	10 (20.0)	0.140	13 (21.0)	21 (20.4)	0.929
Psychiatric problems, yes	21 (8.9)	3 (13.6)	2 (4.0)	0.163	11 (17.7)	5 (4.9)	0.007**
Substance use problems, yes	15 (6.3)	1 (4.5)	5 (10.0)	0.660	6 (9.7)	3 (2.9)	0.082

Distant relationship indicates that relationship with family member was not close

^*^p < 0.05

^**^p < 0.01

^***^ *p* < 000.1

**Table 3:** Psychiatric disorders of study subjects at index hospitalization during adolescence, by recurrence of depression by young adulthood

	Total data (n = 237)	Males			Females		
		Recurrent depression (n=22)	Non-recurrent depression (n= 50)	Group difference, *p*	Recurrent depression (n=62)	Non-recurrent depression (n= 103)	Group difference,*p*
Adolescent psychiatric disorders*, n (%)*							
Affective disorders	183 (77.2)	35 (70.0)	15 (68.2)	0.877	82 (79.6)	51 (82.3)	0.677
Substance use disorders	88 (37.1)	21 (42.0)	10 (45.5)	0.785	35 (34.0)	22 (35.5)	0.844
Conduct disorders	85 (35.9)	25 (50.0)	9 (40.9)	0.477	33 (32.0)	18 (29.0)	0.686
Anxiety disorders	67 (28.3)	9 (18.0)	5 (22.7)	0.749	30 (29.1)	23 (37.1)	0.288
Psychotic disorders	16 (6.8)	3 (6.0)	0 (0.0)	0.548	7 (6.8)	6 (9.7)	0.558

^*^p < 0.05

^**^p < 0.01

^***^p < 000.1

**Table 4:** Familial risk factors in childhood and adolescence as predictors for recurrence of depression by young adulthood

Familial risk factors in childhood and adolescence	Likelihood for recurrence in depression by young adulthood					
	Male study subjects			Female study subjects		
	OR	95% CI	p	OR	95% CI	p
*Model 1 (family-related factors)*						
Large family six (five or more siblings)	–	–		4.00	1.51–10.56	0.005**
*Model 2 (perceived characteristics of family members)*						
Distant relationship with mother	2.95	1.01–8.64	0.048*	2.20	1.05–4.62	0.037*
Paternal psychiatric problems	–	–		2.95	1.12–7.73	0.028*
Psychiatric problems of sibling(s)	–	–		4.08	1.30–12.81	0.016*
Model 3 (model 1 + model 2)*						
Large family six (five or more siblings)	–			4.28	1.54–11.93	0.005**
Distant relationship with mother	3.94	1.22–12.77	0.022*	2.23	1.03–4.81	0.042*
Paternal psychiatric problems	–			3.44	1.29–9.17	0.013**
Psychiatric problems of sibling(s)	–			3.72	1.21–12.36	0.032**

The table shows only the results for final models with statistically significant predictors observed in stepwise logistic regression analyses. *Age at admission to adolescent inpatient care was related to recurrent depression in males (OR = 1.15, 95%CI 1.00–2.41), p = 0.049), but not in females

^*^p < 0.05

^**^ *p* < 0.01

^***^ *p* < 000.1

## Results

### Family-Related Factors

Family-related factors, assessed during index hospitalization in relation to recurrence of depression by young adulthood of the study subjects are presented in Table 1. Generally, family adversities were emphasized in those suffering from recurrent depression. However, the only statistically significant difference between study-groups was observed in the family sizes of female participants. A greater proportion of females with recurrent depression came from large families (five or more children) compared to those with non-recurrent depression (23% vs. 7%) (χ^2^ = 8.68, df = 1, p = 0.003).

### Perceived Characteristics of Family Members

Study subject’s perceptions of the closeness of their relationship with their family members (mother, father, siblings), and their perceived psychiatric and substance use related problems of family members in association to recurrence in depression are outlined in Table 3. The results showed that males reporting having a distant relationship with their mother were more likely to have diagnosis of recurrent depression by young adulthood, compared those in the non-recurrent depression group (45% vs. 22%) (χ^2^ = 4.07, df = 1, p = 0.044). In females, psychiatric problems of fathers (21% vs. 9%) (χ^2^ = 5.01, df = 1, p = 0.025) and siblings (18% vs. 5%) (χ^2^ = 7.341, df = 1, p = 0.007) were related with recurrent depression.

### Psychiatric Disorders During Adolescence

Table 3 shows psychiatric disorders diagnosed during adolescent psychiatric inpatient care. The most common disorders during adolescence were affective (77%), substance use (37%) and conduct (36%) disorders. The prevalence of these adolescent psychiatric disorders did not statistically significantly differ between the recurrent and non-recurrent depression groups, either in male or female study subjects.

### Family-Related Predictors for Recurrence in Depression

Three logistic regression models were performed to investigate the deeper the role of familial risk factors in the recurrence of depression in the study subjects (Table 4). In the final model 3, when all familial risk factors were simultaneously entered into the stepwise logistic regression analysis, an increased likelihood for recurrence in depression was associated to a distant relationship with the mother both in both male (OR = 3.9, p = 0.022) and female (OR = 2.2, p = 0.042) subjects. Further, in females, psychiatric problems in fathers (OR = 3.4, p = 0.013) and siblings (OR = 3.7, p = 0.032) as well as large family size (OR = 4.3, p = 0.005) were all associated to an increased likelihood to develop recurrent depression by young adulthood. None of the adolescent psychiatric disorders (see Table 3) was associated to the recurrence of depression of the study participants.

## Discussion

We examined the association of several familial risk factors in childhood and adolescence to recurrence of depression, by young adulthood, in former adolescent psychiatric inpatients, diagnosed with depression during their lifetime. Our major finding was the importance of a close maternal relationship as a protective factor against the offspring developing recurrent depression. A distant relationship with the mother was shown to increase the likelihood for recurrence of depression by up to 2-fold in male, and up to 4-fold in female subjects. There may be multiple possible reasons for this observed association. One way to approach this is from an evolutionary-psychologic perspective, in which the child, who is devoid of maternal care and guidance, develops flawed or inadequate emotional coping mechanisms. Positive mental well-being in later life is associated with good parental care and less parental psychological control [30]. Supporting this hypothesis is the finding that poor mother-child bonding is associated with internalizing problems in the offspring [31, 32]. A distant mother can also leave the child at risk of experiencing adverse childhood experiences (ACEs), which, in turn, can elevate the risk of the child suffering from depression [33]. Conversely, the reverse is also possible, in that a cold, distant relationship may be a result of factors other than ACEs, which remained beyond the analyses of our study.

Our findings are, in most part, in line with findings of previous studies and our original hypothesis. Although no single socio-economic factor statistically significantly differentiated the study subjects with non-recurrent and recurrent depression, the overall view was that the study subjects developing recurrent depression later in life had grown up in lower socio-economical settings and, presumably, in more stressful environments. This is in line with previous literature, indicating that lower parental education, employment rate and familial social status are associated with higher incidences of depressive episodes in the offspring [34–36]. There are two proposed theories to explain this association. According to the stress theory, people with higher socio-economic status are in better possession of personal resources, such as coping style or self –esteem, which protect against depression [34]. Alternatively, the strain theory focuses on the impact of community features such as values, social welfare, social cohesion, infrastructure, and public health policy in developing depression [34]. Thus, it is justifiable to conclude that the development of recurrent depression is a result of many different factors acting in unison. Further, in clinical practice, when assessing a youth’s living conditions, it is important to assess the situation holistically, rather than focussing on specific separate family environmental factors.

In our study, female patients who had reported paternal psychiatric problems were at an over 3-fold increased likelihood of developing recurrent depression. The literature seems to agree that parental psychopathology increases the risk of mental health problems in the offspring [37]. Previous research is mainly focused on the impact of maternal mental health conditions on the offspring’s well-being, while corresponding research of the impact of paternal mental health problems on the offspring has been relatively scarce. Our findings are in agreement with a systematic review on paternal depression by Sweeney and MacBeth [38], showing a pattern of associations between paternal depression and increased risk of internalizing and externalizing behaviour in the offspring. The review reported that the association was modest, but consistent, from childhood into young adulthood. Furthermore, the risk was proposed to be mediated by indirect mechanisms, such as impaired father-child-relationships, development of insecure attachment patterns, reduced father interaction and involvement, and increased marital conflict.

Another important finding of our study was the impact of large family size on recurrent depression in females. One explanation for this result may be that offspring in larger families tend to get less attention and individual care, thus the relationship between the child and parents may feel more distant. Earlier studies [39, 40] have indicated that large family size is related to various mental health problems (such as suicidality, self-harm or non-schizophrenic psychoses) in the offspring. Our finding is an important addition to previous literature since, to our knowledge, studies examining the association between recurrent depression and large family size are mainly lacking.

In large families the importance of relationships between siblings also grows, as the presence of parents diminishes. In their meta-analysis, Buist and workgroup reported that more sibling warmth, less sibling conflict and parents showing equal care towards their offspring were all significantly associated with less internalizing and externalizing problems [41]. This concurs with our finding that perceived psychiatric problems of the siblings were related to the risk for recurrent depression, particularly, among female study subjects. Sibling’s psychiatric problems may be a result of the same defective parenting patterns the offspring has been subjected to. One thing to keep in mind is that, in many cases a large family can also act as a protective factor if the child gets enough support and care from the parents. Offspring of families with two or three children have actually reported to have less psychiatric problems than single-born children [40]. We are also aware that birth order of an individual can bring an independent effect into this phenomenon. In our study, the percentage of middle-born children was higher among female study subjects with recurrent depression in comparison to single-episode depressed females. This finding deserves further studies to explore this phenomenon.

In our study, accumulation of adverse familial risk factors was emphasised in female compared to male subjects. It could be theorized that adolescent girls are more likely to be subjected to, or react more readily, to intra-familial stress and the mood inside the family compared to boys. Earlier literature supports this hypothesis [42, 43]. This finding also fits well with the social role theory, according to which females are thought to adopt a more communal and interpersonal facilitative behaviour [44], and the stress exposure (SE) models that suggest that girls tend to experience more (meditational SE-model), or react more strongly (moderational SE-model), to social stressors than boys and, consequently, have more internalizing symptoms such as depression in comparison to boys [43].

Contrary to earlier studies, our study did not reveal any significant associations of psychiatric disorders diagnosed in adolescence to later recurrence in depression of the study subjects. For example, in a Danish population-based cohort study, it was found that recurrent depression was more likely in participants with co-morbid anxiety disorders, in comparison to control population suffering from single episode depression [45]. However, our finding is consistent with the meta-analysis of Burcusa and Iacono, showing that an adolescent’s comorbid psychopathology does not increase the risk for recurrence in MDD as it does in adults [26].

Despite the significant changes occurring in society, the problems faced by adolescents today are fundamentally the same as those faced by previous generations. What has changed, however, is the number of psychiatric hospital beds, which has been in steady decline in Finland over the last 20 years. Today, patients are more likely to be treated in outpatient care, and the time spent in psychiatric hospital care has been reduced [46]. Another observation, based on our clinical experience, is the increased number of substance abusers among our adolescent patients. The findings of and adolescent study compared child and adolescent psychiatric inpatients in Finland between the years 2001 and 2011 reported that a growing percentage of patients were female, suffering from depression- and anxiety-scale disorders, and the share of adolescent patients with poor general functioning (CGAS-score of 1-30) had increased while treatment times had decreased [47].

### Strengths and Limitations

A strength of our study is that psychiatric diagnoses at the index period during adolescence were based on the semi-structured K-SADS-PL interview, showing high concurrent validity and inter-rater agreement (93 to 100%) [28, 48]. Further, the quality of the data from the Finnish National Care Register for Health Care, provided by the Finnish of Health and Welfare, has been shown to be high and provides access to comprehensive information about the use of national inpatient or outpatient health care resources at a national level [49]. In addition, by examining parental problems, as experienced by the study subjects during their adolescence, it was possible to obtain a more comprehensive and inclusive insight into each patient’s family life than we perhaps would have obtained had we just used information on parental diagnoses of psychiatric or substance use disorders. It also better represents the child’s feelings within their family and asks whether they find that their parents’ behaviour to be problematic or not [50]. We considered the possibility of patients under reporting family mental health problems, given that mental health and substance use problems are, in many cases, still taboo in our society. An important strength in our study is also its longitudinal nature, with a long follow-up period of 9 to 14 years after discharge from adolescent psychiatric inpatient care. It is noteworthy that this period mainly covers adolescence and early adulthood of the study subjects, the period of time when the onset of depression is most likely to occur. This gives us a unique insight into the developmental process of recurrent depression in young adults. Finally, the study population was very homogenous and selection bias can be assumed to have been small, given the high participation rate (83.7%).

The following limitations of our study are acknowledged. Because our study population consisted of former adolescent psychiatric inpatients, these findings are not directly generalizable to the general Finnish population of the same age. Another limitation is that, from our register-based data, it was impossible to determine whether or not the patient achieved complete recovery before a new treatment period for depression. For the purposes of this study, we considered an 8 week minimum period between subsequent treatments for depression, to indicate a ‘symptomless period’ as is also suggested by the ICD-10 criteria. In addition, we have no information on the quality and length of outpatient treatment offered to the adolescents after their first episode of depression, and whether they had access to individual psychotherapy or family therapeutic approaches. Our statistical findings do not allow making conclusions which of the adolescent-related predictors had major impact on depression recurrence by the young adulthood. It is assumable that some potential risk factors for depression recurrence will relate to later lifer of the study participants, and in this respect, our database is limited.

Since our data was collected in the early 2000s, it may be that the field of adolescent psychiatry care has somewhat changed over the time and, therefore, is remains unknown whether this change could have an impact on our results. Our current society and adolescent lifestyle is now much more interconnected across the globe, and the rise of social media may have created a new environment that can potentially affect adolescent mental health adversely [51]. A previous study, published using the same population as our study sample, reported that an interest in computers and video games did not increase the risk of any specific psychiatric disorder among participants but, conversely, a decreased likelihood of a substance-related disorder was observed in boys with computers as a hobby [52]. The authors concluded that social contacts and peers play an important role in preventing adolescent depression, a statement we can also whole-heartedly endorse.

## Summary

Young persons diagnosed with a depressive disorder are at increased risk for recurrence in their depression. Therefore, to prevent relapse or recurrence of depressive disorders in children and adolescents is a challenge for clinical practitioners. In our study, we examined which family environmental factors contribute to differences between recurrent and non-recurrent depression in young patients. The initial study sample covered 237 former adolescent psychiatric inpatients with depression, of which 35.4% had developed recurrent depression by the young adulthood. Recurrence in depression was shown to associate to distant maternal relationships and, in particularly in female study subjects, with psychiatric problems of the father and siblings, and having a grand multiparous mother. These results highlight the importance of including the whole family in the treatment of adolescents with depression. Our findings may also help identifying those youths, who are at risk of developing recurrent depression later in their life. This would allow clinicians to focus more efficiently on preventive measures to help those individuals and their families. Our study findings suggest not only a need to develop more robust and effective screening tools, for clinical use, when evaluating the risk of recurrent depression of young patients, but, above all, encourages the use of effective family-centred approaches in treatment of adolescents with depression.
